# Meta-analysis of effects of age on intestinal digestibility of liquid feeds in young calves

**DOI:** 10.3168/jdsc.2020-0057

**Published:** 2021-03-12

**Authors:** J.D. Quigley, T.S. Dennis, F.X. Suarez-Mena, T.M. Hill, K.M. Aragona

**Affiliations:** Nurture Research Center, Provimi North America, Cargill Animal Nutrition, Brookville, OH 45309

## Abstract

A meta-analysis was conducted to determine the effect of age on digestibility of liquid feeds fed to preweaned Holstein calves. Calves were 4 to 76 d of age and fed whole milk or milk replacer containing all-milk protein. Least squares means of apparent total-tract N, DM, and fat digestibilities (n = 84, 51, and 24, respectively) from 12 publications were used. Broken line regression and mixed model ANOVA were used to determine ages at which digestion of nutrients no longer increased with age, indicating maturation of digestive capacity. Digestibility of DM, N, and fat reached maximum digestibilities of 94.1, 92.7, and 97.5% at 29, 29, and 32 d of age, respectively. These data indicate age-related changes in digestion of liquid feeds in preweaned Holstein calves and allow correction of apparent total-tract digestibility estimates in nutrient supply models. Digestibility of liquid nutrients in calves fed whole milk or all milk containing milk replacers reached maximal capacity at approximately 30 d of age.

## Highlights

• Intestinal digestibility of nutrients in milk and milk replacer is immature in young calves.
• Digestibility increases with advancing age and maturation of the gastrointestinal tract.
• Digestibility of dry matter, nitrogen, and fat increased to approximately 30 days of age, after which no further improvement was observed.

## Graphical Abstract

**Figure:**
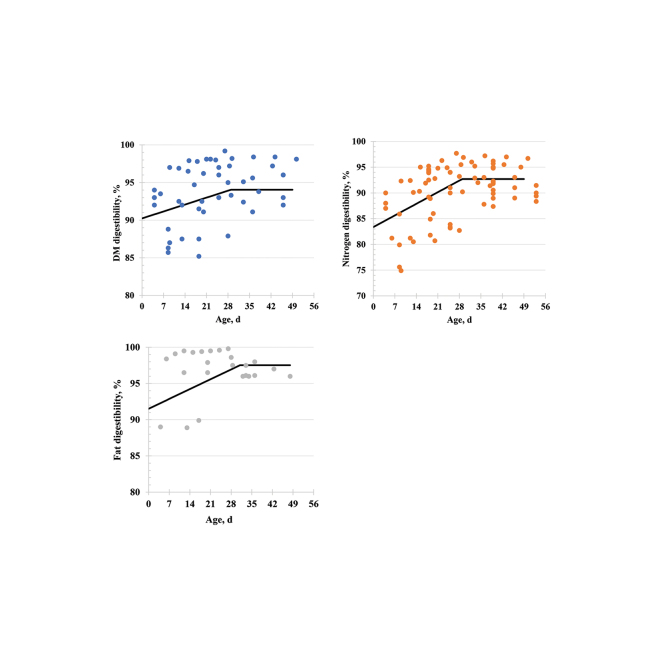
**Summary:** Intestinal digestibility of nutrients in milk and milk replacer is immature at birth but increases with advancing age. We showed that digestibility of dry matter, nitrogen, and fat increased to approximately 30 days of age.

Liquid feeds fed to calves before weaning are normally shunted past the reticulorumen via nervous closure of the esophageal groove, thereby avoiding ruminal fermentation of nutrients. Therefore, the maturity of gastric and intestinal digestion determines nutrient digestibility of these feeds. Numerous studies have documented effects of age on digestibility of nutrients in liquid feed for preweaned dairy calves. For example, Terosky et al. (1997) reported that N digestibility in calves fed milk replacers containing whey or skim milk proteins increased from approximately 70% to 90% from 2 to 8 wk of age. Others reported similar increases with advancing age, generally concluding that digestibility increases to approximately 3 wk (Arieli et al., 1995; Terosky et al., 1997; NRC, 2001) or 5 wk (Guilloteau et al., 2009) of age. Total nutrient digestibility and rate of change in nutrient digestibility with advancing age may also vary with ingredients in the feed, as non-milk proteins are digested differently than milk proteins (e.g., Branco-Pardal et al., 1995; Thornsberry et al., 2016). Also, fat digestibility is affected by fat source (Radostits and Bell, 1968; Johnson and Leibholz, 1980) and method of processing and emulsification (Hopkins et al., 1959; Jenkins et al., 1981).

Nutrient supply models generally ignore the maturation of intestinal digestibility with advancing age (e.g., NRC, 2001). Unfortunately, few published data document changes in digestibility with advancing age or protein source. Therefore, the objective of this research was to develop a model of changing nutrient digestibility of liquid feeds in calves to weaning.

Published research studies that measured apparent total-tract digestibility of nutrients of milk or milk replacer in preweaned Holstein calves were identified (Table 1). The initial literature search was conducted using Google Scholar and PubMed using the search terms “calf,” “calves,” “digestion,” “digestibility,” “milk,” and “milk replacer.” Criteria for inclusion were measurement of apparent total-tract digestibility of DM, N, or fat in Holstein calves fed whole milk or milk replacer on at least 2 measurement periods. Trials were excluded if significant dry feed was consumed or when novel ingredients or non-milk proteins were included in milk replacer treatments. Treatments in which calves were reported to have experienced diarrhea were also excluded.

**Table 1:** Raw means from studies comprising the data used in model development

Reference	No.^1^	Type	Age, d	Digestibility		
				N, %	DM, %	Fat, %
Noller et al., 1956	7	Milk	12–36	81–93	88–96	97–99
Wood et al., 1970	6	Milk	9–19	80–89	85–92	
Volcani et al., 1971	2	Milk	34–48	92–95		96
Bouchard et al., 1973	4	Skim	9–38	75–92	87–94	
Toullec et al., 1974	4	Milk	17–76	92–96	95–97	89–97
Donnelly and Hutton, 1976	18	Milk	18 and 39	89–96		
Neergaard, 1979	6	Milk	15–50	98–98		
Russell et al., 1980	8	Milk	6–27	81–98	94–99	98–99
Akinyele and Harshbarger, 1983	4	Milk	10–33	90–95	88–95	89–98
Stobo, 1983	12	Skim	4–46	87–94	92–97	
Terosky et al., 1997	11	Skim/WPC^2^	25–53	77–92		
Guilloteau et al., 2009	2	Skim	4 and 32	87–96		89–96
Total or range	84		4–76	75–98	85–99	89–99

1 Number of least squares means per study.

2 Whey protein concentrate.

A broken-line regression approach (Robbins et al., 2006) was used to analyze the data, assuming that digestibilities increased to a point of relative maturity within the ages included in the database. The NLMIXED procedure of SAS (version 8; SAS Institute Inc.) was used. Random component for asymptote was included in each analysis, and treatment within study was included in the random statement as subject.

Following determination of age at which maximal nutrient digestion was achieved, the data set was limited to measurements ≤30 d and was subsequently modeled using the mixed modeling approach described by St-Pierre (2001). The model was nutrient digestibility (%) = age (days); intercept and age were included as random components, and treatment was included as subject.

Digestibility data were available from 12 studies, with 30 unique treatments and a total of 84, 51, and 24 observations for N, DM, and fat digestibilities, respectively (Table 1). Age of calf ranged from 4 to 76 d, and digestibilities of N, DM, and fat ranged from 75 to 98, 85 to 99, and 89 to 99%, respectively.

Results of broken-line regression and mixed model analyses are in Table 2. For the broken-line regressions, asymptotes for DM, N, and fat were 94.1, 92.7, and 97.5%, respectively. Ages at which mature digestion was reached were 29, 29, and 32 d, respectively. Broken-line regressions for N, DM, and fat digestibility are in Figure 1.

**Table 2:** Results of broken-line and mixed model analysis of nutrient digestibility for DM, N, and fat in liquid feeds fed to preweaned Holstein calves

Digestibility^1^	No.	Broken-line statistics^2^							No.	Mixed model statistics^3^				
										Intercept		Slope		R^2^
		Asymptote	SE	Slope	SE	Breakpoint	SE	Adj R^2^		Coefficient	SE	Coefficient	SE	
DM	51	94.05	0.951	−0.134	0.0358	29.00	5.239	0.86	37	90.153	1.1607	0.137	0.0400	0.38
N	84	92.70	0.881	−0.323	0.0652	28.91	3.233	0.72	50	83.237	1.7071	0.337	0.0710	0.41
Fat	24	97.52	1.137	−0.194	0.0518	32.00	6.198	0.74	16	91.842	1.8837	0.155	0.0626	0.48

1 Apparent total-tract nutrient digestibility.

2 Asymptote = digestibility (%) at which maximal digestibility occurred; Slope = slope (%) of first segment of broken-line regression; Breakpoint = age (d) at which no further increase in percent digestibility occurred; Adj R^2^ = adjusted R^2^ of regression calculated as described by Robbins et al. (2006).

3 Mixed model analysis of apparent total-tract digestibility of DM, N, and fat from 1 to 30 d of age. All coefficients were significant (*P* < 0.001).

**Figure 1:**
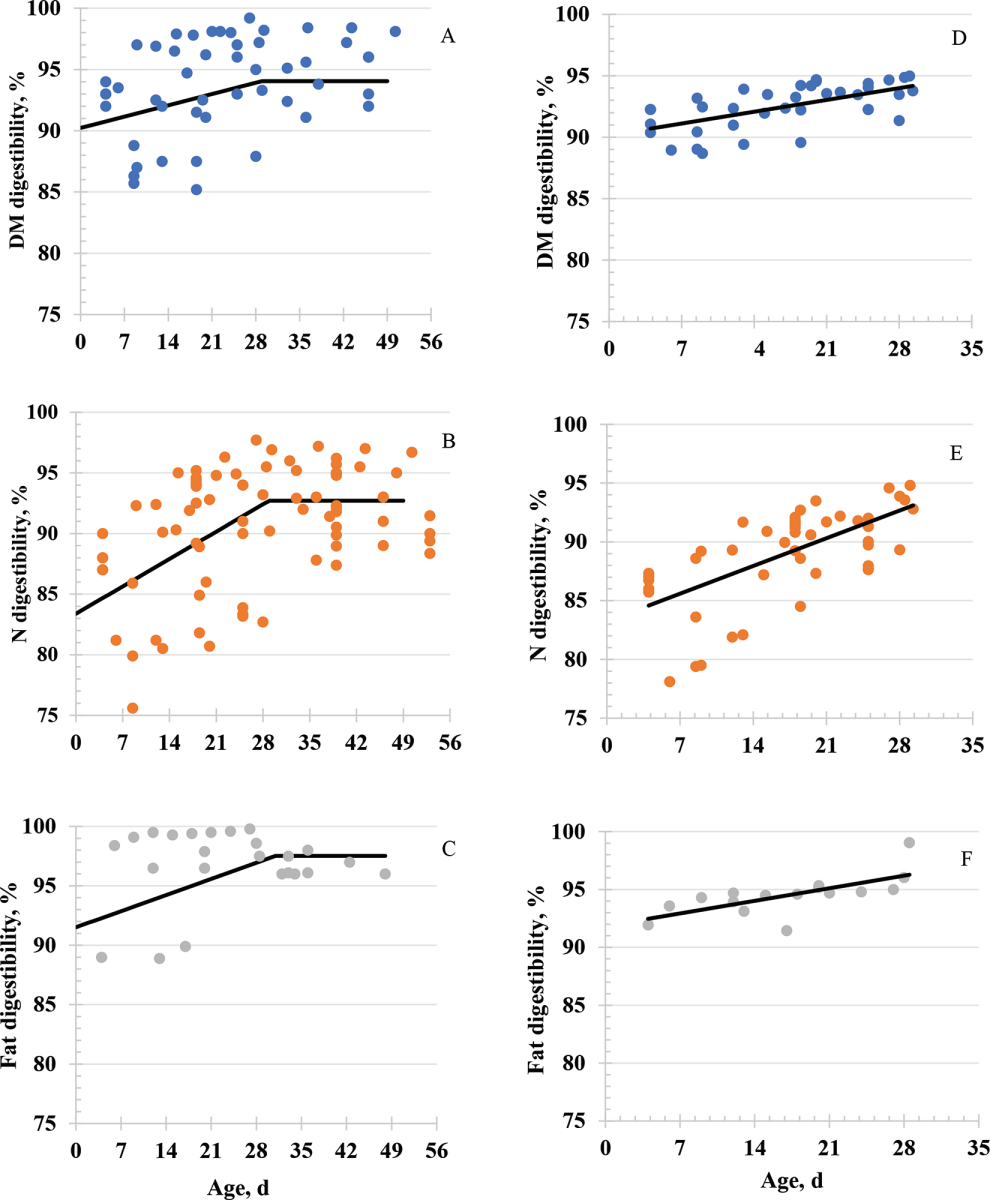
Broken-line mixed model analyses for DM (A), N (B), and fat (C), and mixed model linear regression to 30 d of age for DM (D), N (E), and fat (F) apparent total-tract digestibility in Holstein calves fed liquid feeds containing all milk. Mixed model regression observations are adjusted means as described by St-Pierre (2001).

Mixed model linear regression coefficients (Table 2) for digestibilities to 30 d of age were similar to calculations for broken-line regression. The coefficients of determination were calculated by linear regression of adjusted data on age (St-Pierre, 2001). The intercepts for fat and total DM were greater than 90%, and the intercept for N digestibility was 83%. Slopes indicated relative rate of digestive maturation from 4 to 30 d of age. Graphical representations of the change in nutrient digestibility from 1 to 30 d of age for DM, N, and fat are shown in Figure 1D, E, and F, respectively.

Knowledge of changing nutrient digestibility with maturation of the gastrointestinal tract in young calves is essential to accurate formulation of diets to meet nutrient requirements. Numerous studies have documented changing digestion of nutrients with advancing age (Noller et al., 1956; Terosky et al., 1997; Guilloteau et al., 2009), though others have reported no significant change in digestibility of fat or N between 8 and 28 d (Strudsholm, 1988). Changes in enzymatic activities and gastric and pancreatic secretions responsible for changing nutrient digestibility in the first month of life have been reviewed (Ruckebusch et al., 1983; Thornsberry et al., 2016).

Digestibility of DM increased from 90% at d 4 to 94% on d 30 with no increase in digestibility thereafter. Apparent DM digestibility of milk and calf milk replacer has been reported to be approximately 95% (Branco-Pardal et al., 1995) in calves older than 30 d of age, which is similar to the asymptote value of 94%.

Estimated digestibility of N for whole milk and all-milk-protein calf milk replacer ranged from 83% at 1 d of age to 93% at 29 d of age. The value of 93% N digestibility is identical to the estimate used by NRC (2001) to calculate apparent digestible protein from CP.

Digestibilities of N early in life were lower as a proportion of mature N digestibility, which may be related to the relative immaturity of the gastrointestinal tract and pancreatic secretions (Guilloteau et al., 2009). However, the rate of increase of N digestibility with age was greater than that for other nutrients, suggesting that maturation of protein digestion is rapid. Liang et al. (2016) reported that N digestibility in Jersey calves fed 11.6 g/kg of BW of a 20% CP, 20% fat milk replacer containing all milk protein increased from 80.7% at 1 to 3 d of age to 86.7% at 4 to 7 d of age. Conversely, N digestibility did not change with age when calves were fed 19.2 g/kg of BW of a 28% protein, 20% fat milk replacer (90.1 and 87.1% N digestibility during 1 to 3 and 4 to 7 d of age, respectively). Similarly, Quigley et al. (2019) recently reported no significant increase in protein digestibility from 1 to 3 wk of age when calves were fed 0.66 or 0.77 kg/d of milk replacer; however, protein digestibility values were generally low (83–88%) during the trial.

Only 6 studies reported apparent total-tract fat digestibility that met all criteria for inclusion in the metanalysis. Digestion of fat increased with increasing age to approximately 32 d (Figure 1C) to maximum apparent total-tract digestibility of 97.5% (Table 2). However, 3 least squares means ≤90% appeared to influence the regression (Figure 1C). Increasing production of pancreatic lipase in the first month of life may contribute to increasing digestibility of lipid by the milk-fed calf (Guilloteau et al., 2009). Use of emulsifiers and processing technology has also improved digestibility of lipids in commercial milk replacer formulas (Thornsberry et al., 2016). On the other hand, Quigley et al. (2019) recently reported that fat digestibility averaged 96% at 1 wk of age when calves were fed high-quality commercial milk replacer containing lard as the primary fat source.

Broken-line regression indicated that digestive maturity occurred at 29, 29, and 32 d of age for DM, N, and fat, respectively. A mixed model approach was also used for data from 1 to 30 d of age (Table 2; Figure 1D, E, and F). Results were similar to those for broken-line regressions.

Changing digestion of nutrients during the first month of life influences total nutrient supply to the animal and can influence growth. Often, models of nutrient supply overestimate growth of calves during the first month of life, which may be related to overestimated supply of energy and protein. Adjusting digestibility of liquid feeds using the equations developed herein may improve prediction of nutrient supply and estimates of growth in young calves.
